# Topical Brazilian propolis improves corneal wound healing and inflammation in rats following alkali burns

**DOI:** 10.1186/1472-6882-13-337

**Published:** 2013-11-27

**Authors:** Luiz Fernando Taranta Martin, Eduardo Melani Rocha, Sérgio Britto Garcia, Jayter Silva Paula

**Affiliations:** 1 Department of Ophthalmology, Otorhinolaryngology and Head and Neck Surgery, School of Medicine of Ribeirão Preto - University of São Paulo, Av. Bandeirantes, 3900 – 12°. Andar, Ribeirão Preto, São Paulo, Brazil; 2 Department of Pathology, School of Medicine of Ribeirão Preto - University of São Paulo, São Paulo, Brazil

**Keywords:** Brazilian propolis, Alkali burn, Cornea regeneration, Corneal inflammation, Ki-67

## Abstract

**Background:**

The objective of this study was to investigate the effects of the Brazilian *Scaptotrigona sp* propolis, a widely used folk medicine, in corneal wound healing and inflammation.

**Methods:**

Corneal epithelial defects of 1 mm in diameter were made in the right eyes of Wistar male adult rats by cauterization with silver nitrate sticks. Subsequently, they were divided in two groups (n = 40 rats/group): Brazilian propolis (BP) group was topically treated with a microemulsion containing 1% Brazilian propolis; vehicle (VH) group received the same formulation without propolis. The epithelial defect area was photographed and measured at t = 0 (wound induction), and after 12, 24, 48 and 120 h of treatment. The inflammatory response was evaluated based on counting of neutrophils. Epithelial regeneration rates were determined based on Ki-67 expression in basal epithelial cells. Comparisons were made using the Kruskal-Wallis and the Mann–Whitney U test.

**Results:**

The BP group presented both smaller epithelial defect areas at 12, 24 and 48 h and fewer corneal infiltrating neutrophils at 24 and 48 h (*P* < 0.01) than the VH group. These effects were associated with more pervasive Ki-67 staining in the BP group at 12 and 24 h (*P* < 0.05).

**Conclusions:**

Topically applied BP accelerated wound healing and reduced the inflammatory response to silver nitrate-induced corneal alkali burns in rats.

## Background

The corneal epithelium plays important roles in the maintenance of corneal function and integrity. Corneal epithelial defects resulting from corneal injury such as a chemical burn may heal inappropriately and lead to corneal opacification, neovascularization, infection, and visual loss [1]. Although patients with these complications are frequently seen in eye clinics and the molecular mechanisms to revert them are known from translational studies, there are no commercially available products to effectively treat delayed corneal re-epithelization without inducing adverse side effects [1,2].

Herbal therapy constitutes the largest proportion of the anecdotal complementary and alternative medicines used in the United States of America, accounting for more than 15 million consumers [3]. Such increases in their usage necessitates that their safety and efficacy should be more extensively evaluated by employing accepted scientific criteria for this purpose.

Propolis (honeybee glue) is a resinous mixture of botanical balsams and resin with digestive enzymes of bees used as a sealant in the hive. It is currently used as a dietary supplement for the treatment of various diseases [4-7]. Indeed, it has been shown to have a wide range of biological activities, attributable to the presence of flavonoids and caffeic acid phenethyl ester [4]. Hence, the putative therapeutic properties of propolis could be related to its antibacterial [5,6], anti-inflammatory [7] and antioxidant activities [8,9]. A previous study has suggested that red propolis, a kind of propolis commonly used in Eastern Europe, reduces corneal inflammation [10].

Propolis has a variety of botanical sources, and its chemical composition can also be variable. Several different Brazilian propolis preparations are commonly used to supplement some foods and beverages. There are indications that they maintain or even improve human health [11,12]. Even though there are anecdotal indications that the topical application of native Brazilian stingless bee propolis *(Scaptotrigona sp)* to a penetrating corneal injury promotes corneal wound healing, and reduces inflammation, its effects have not been rigorously evaluated in any experimental model. The current study evaluated its effect on corneal inflammation and wound healing in rats after alkali injury.

## Methods

### Animals and procedures

All experimental procedures were approved by the Ethics and Animal Experiments Committee at the University of São Paulo, Ribeirão Preto Medical School. Animals were treated in accordance with guidelines provided in the ARVO Statement for the Use of Animals in Ophthalmic and Vision Research.

Wistar male rats 250–300 g (n = 80), housed in the Central Bioterium of the University of São Paulo (Ribeirão Preto Campus), were anaesthetized with halothane (4%, in air), and had the centers of their right corneas cauterized with a silver nitrate applicator stick (75% silver nitrate, 25% potassium nitrate; Graham-Field Inc, Hauppauge, NY). The applicator was held in contact with the cornea for 2 s, producing a discrete grayish-white lesion of 1 mm in diameter. The cauterized eye was then rinsed several times with room temperature saline. Rats recovered fully from anesthesia within a few minutes, and exhibited no outward signs of distress.

After wounding, animals from a subset of each group (n = 40 rats/group) were examined with a slit lamp. A drop of fluorescein was used to stain the damaged area and to measure the lesions. The rats were held in the focus position by a fixation device commonly used for stereotaxic surgical procedures, attached to a plastic base. Photographs were taken at five time points: immediately after lesion-induction (time 0), and 12, 24, 48 and 120 h post-injury. Animals were labeled on the inferior eyelid, with a paper designating their group, number and time after lesion. This paper also contained a 1 mm ruler for posterior measurement of the corneal defect areas and conversion from pixels to millimeters.

In the slit lamp, cobalt blue light and a zoom of 16 times were used (Carl Zeiss - Ltda, Germany). Photographs were taken with a digital camera (DSC-W5, Sony Ltda, Japan) connected to the slit lamp by an optical system (D.F. Vasconcelos, Brazil). Images were downloaded to a desktop computer and corneal wound areas were measured, using NIH Image J 1,33u software (NIH, USA).

### Propolis preparation and use

Brazilian propolis (BP) used is produced by *Scaptotrigona sp*. and was obtained from the Northeast region of Brazil (Barra do Corda, MA, Brazil). These bees are stingless, highly eusocial, less harmful to humans and more resistant to diseases than *Apis mellifera*[13].

The propolis extract used in the present work was obtained directly from beehives using water and ethanol 70% (7:3). A BP microemulsion was prepared containing 1% of propolis dry matter in the final product which was solubilized in a mixture of polyethylene glycol-6- caprylate/caprate, polyglyceryl-6-dioleate, glycerides caprylate/caprate (10,0%) (Mackaderm MicroExpress - McIntyre, USA), chloride benzalcone (0.01%) and deionized water 100% per system MilliQ (Millipore, USA).

The vehicle (VH) used as placebo (control group) was also prepared using polyethylene glycol-6- caprylate/caprate, polyglyceryl-6-dioleate, glycerides caprylate/caprate (10,0%) (Mackaderm MicroExpress - McIntyre, USA), chloride benzalcone (0.01%) and water distilled deionized 100% per system MilliQ (Millipore, USA).

Subsequently, BP and VH were filtered separately using a filtering membrane of 0.22 μm pore size (Milipore, MA, USA) and stored in eyedropper polyethylene bottles. Eye drop manipulations were made in sterile conditions, using a laminar flux camera irradiated with ultraviolet light (MiniFlow 1 – Marconi, São Paulo, Brazil). All material was autoclaved before use at 121°C, for 15 min.

Both, the BP and VH groups received two 40 uL eye drops, 10 seconds apart, immediately after corneal cauterization. Treatment was applied four times a day until the rats were euthanized after 12, 24, 48 and 120 h (n = 10/subgroup per time point).

### Tissue processing and data analysis

At each time point, animals were anesthetized and corneas were examined with the slit lamp. After being photographed, still under influence of anesthesia, the rats were euthanized in a carbon dioxide chamber. Corneas were harvested, fixed with formalin, and blocked with paraffin. Histological cuts of 5 μm were performed, transferred to slides, and stained with Hematoxylin and Eosin (HE).

**Figure 1:**
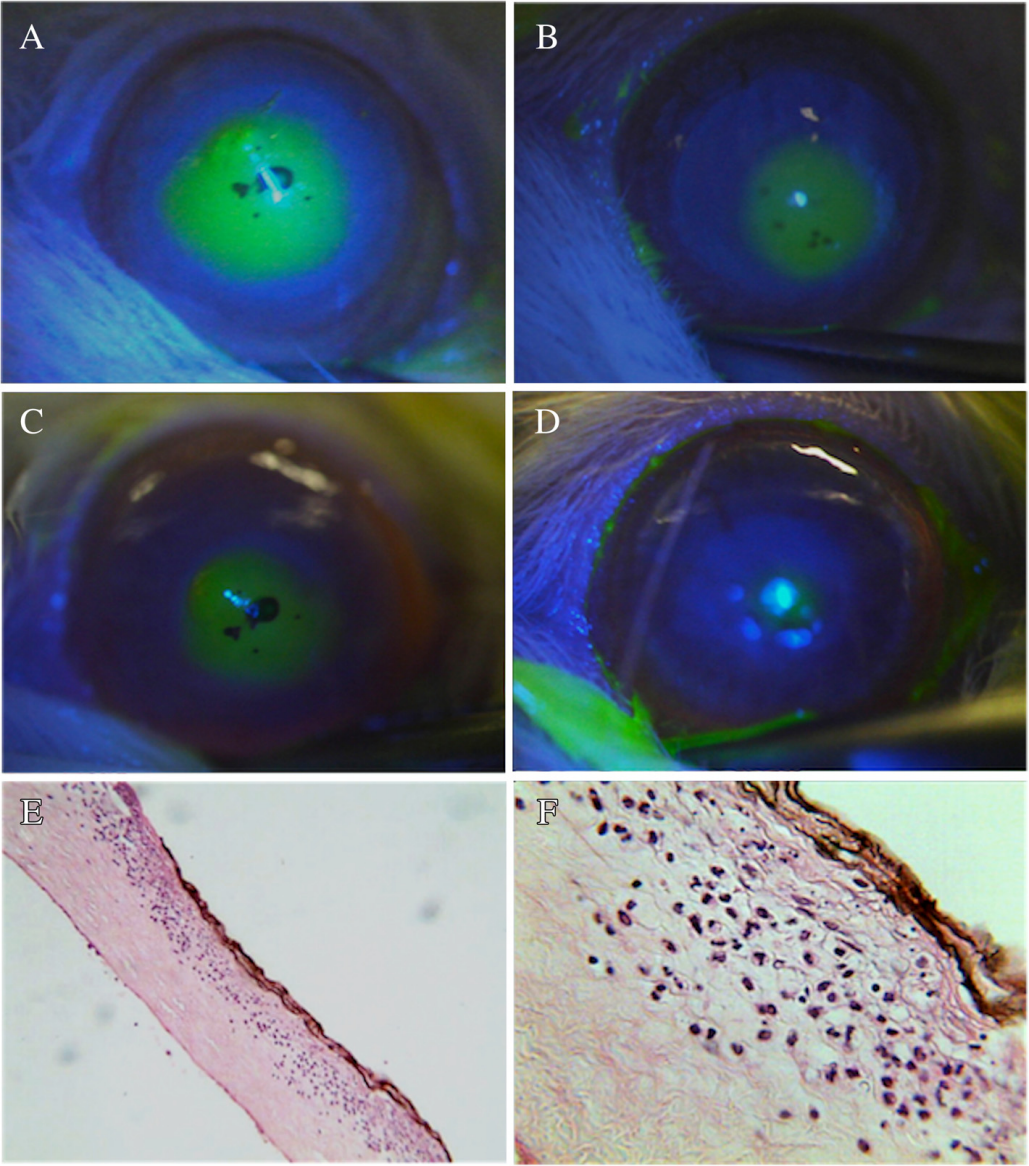
**Photograph composition of representative images of corneas from rabbits of the Brazilian propolis (BP) and vehicle (VH) groups. A **to **D** - comparison of the fluorescent-stained area of injured cornea between groups at different time points, as following: **A** and **B** - photographs at 12 hours of animals from VH and BP groups respectively; **C** and **D** – photographs at 48 hours of animals from VH an BP groups respectively. Note the smaller areas of injury with BP treatment (images **B** and **D**). Image E displays a representative histological section of central cornea of BP group at 24 hours (H&E; original magnification 100X), and **F** shows details of this area, including a brownish central lesion with loss of epithelium and superficial stroma, necrosis and infiltration of neutrophils (H&E; original magnification: 400X).

Slides were analyzed with an optic microscope and at 400x magnification (Olympus BX40 light microscope, Olympus Corporation, Tokyo, Japan) and pictures were taken with a digital camera (Olympus Q-color 5). Three fields were used for cell counting at 400X magnification by an experienced pathologist (SBG), with posterior calculation of the number of cells per mm^2^ (Figure 1). The results presented represent the sum of cells of all three counting fields. The procedure was performed for two slides from the center of paraffin block from each cornea harvested (per time point). The number of neutrophils in the fields was then counted for the four time points (i.e., 12, 24, 48 and 120 h, n = 10/group per time point).

Immunohistochemical analysis of Ki-67 protein expression was used to evaluate basal cell proliferation. In brief, paraffin was removed from the slides throughout re-hydration, endogenous peroxidase was blocked with PBS, and nonspecific antibody binding was blocked with normal horse serum. IgG anti rat Ki-67 was used as primary antibody, diluted 1:200 (Novocastra, Newcastle Upon Tyne, UK) followed by incubation with a secondary antibody (Vectastain Elite ABC kit Universal, Vector, CA, USA), immunoperoxidase with streptavidin/biotin, and DAB (NovoLink, Novocastra, Newcastle Upon Tyne, UK) and counter-staining with HE. Afterwards, slides were mounted and, using the same optical microscope mentioned above, the number of epithelial cells with brown stained nuclei in the corneal basal layer was determined in five serial sections of samples from each animal. As there were slight variations in staining intensity among the different histological sections, only the positively stained cells in the bottom epithelial layer of the outgrowths were evaluated.

### Statistical analysis

Data are expressed as means ± SEM. Comparisons were made using the Kruskal-Wallis test for continuous data comparing several time points, and the Mann–Whitney U test for continuous data comparing BP and VH parameters at each time point (Graphpad 5.0 software, Prism, San Diego, CA). The level of significance used was *P* < 0.05.

## Results

### Wound healing

Digital analysis of the corneal injuries areas stained with fluorescein was used to calculate the size of the wounds (Figure 1). At 12, 24 and 48 h post-injury, the wound defect areas was consistently smaller in the BP than in the VH treated groups (*P* < 0.01) (Table 1). On the other hand, there was no difference between the two groups immediately after making the wounds. At 120 h after injury, in both groups the corneal epithelial defects of all animals had undergone complete re-epithelization (Figure 1).

**Table 1:** Area of corneal epithelial defect at various time points after injury on groups BP (Brazilian propolis) and VH (vehicle) (mean ± SEM)

**TIME (h)**	**VH (mm**^**2**^**)**	**BP (mm**^**2**^**)**	***P***
*0*	0.0389 ± 0.0017	0.0341 ± 0.0014	0.1978
*12*	0.0291 ± 0.0012	0.0160 ± 0.0030	0.0032
*24*	0.0163 ± 0.0021	0.0076 ± 0.0022	0.0007
*48*	0.0075 ± 0.0009	0.0031 ± 0.0007	0.0019
*120*	0.00	0.00	N/A

NA: Not applicable.

### Neutrophil infiltration

Neutrophil infiltration was used as an index to evaluate the effects of propolis on inflammation. Mainly, in the corneal central anterior stromal layer of both groups, necrosis was seen following injury (Figure 1). At 12 h after injury, the number of neutrophils were the same in both the BP and VH groups.

However, at 24 and 48 h, fewer neutrophils were seen in the group BP (*P* < 0.01). After 120 h, there was no longer a difference between the number of neutrophils between the two groups (Table 2).

**Table 2:** Neutrophils count in corneas at various time points after injury in groups BP (Brazilian propolis) and VH (vehicle) (mean ± SEM)

**TIME (h)**	**VH (cells/field)**	**BP (cells/field)**	***P***
*0*	NA	NA	NA
*12*	150.4 ± 9.0	150.3 ± 10.8	1.0000
*24*	448.6 ± 21.7	354.7 ± 9.7	0.0006
*48*	408.0 ± 16.5	326.7 ± 13.3	0.0014
*120*	82.2 ± 20.0	56.9 ± 10.0	0.3752

NA: Not applicable.

### Immunohistochemistry (Ki-67)

**Figure 2:**
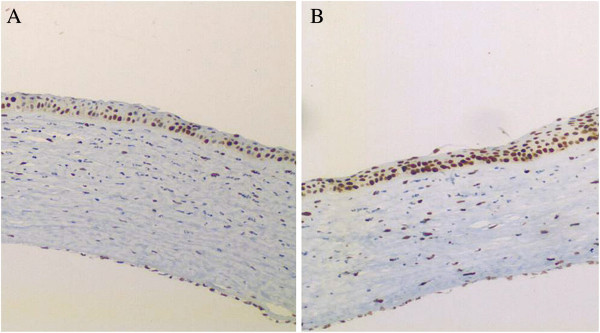
**Immunohistochemical Ki-67 analysis of cornea tissue cauterized with Silver Nitrate, and treated with VH (vehicle - A) or BP (Brazilian propolis - B) eye drops.** Photographs were taken at time-point 12 hours with 400X (original magnification).

There was a higher number of Ki-67 positive cells in the basal epithelial layers in the BP than the VH group at both 12 and 24 h (P < 0.0001 and P < 0.001, respectively) (Figure 2). However, 48 h after injury there no longer was any difference between these groups (Table 3). Furthermore, wound closure occurred in the two groups 120 h after injury, since Ki67 staining was absent, due to complete re- epithelialization.

**Table 3:** Positive Ki-67 stained cells in the corneal basal layer in both groups (VH – vehicle and BP – Brazilian propolis) at the different time point studied (mean±SEM)

**TIME (h)**	**VH (No. of cells)**	**BP (No. of cells)**	***P***
*0*	NA	NA	NA
*12*	46.7 ± 0,02	63.7 ± 0.02	<0.0001
*24*	64.2 ± 0.02	74.15 ± 0.0195	0.0067
*48*	61.7 ± 0.0130	58.05 ± 0.0209	0.1412
*120*	0.0	0.0	NA

NA: Not applicable.

## Discussion

Corneal surface injuries are among the most frequent traumas of the eye [14]. The prompt recovery of such injuries is critical to the maintenance of corneal transparency. Although previous publications [15,16] addressed therapeutic options, no commercial eye drops have been approved for this specific purpose, nor has BP been clinically tested to the best of our knowledge.

Aqueous extracts obtained from BP have antioxidative properties and inhibitory actions on certain enzymes that are greater than those obtained with ethanolic extracts. These extracts are rich in terpenoids and derivatives of coumaric, caffeoylquinic and cinnamic acids, and it is probable that these components have synergistic effects on healing [15-17].

It is known that red propolis causes damage to the corneal epithelial cells of rats when used at concentrations of 7.81 mg/mL [18]. Thus, although there are no specific studies on corneal toxicity of BP, at a concentration of 1% (10 mg/mL) there was no indication in this study that it had any toxic effects.

The mechanisms by which propolis elicits healing effects have been the focus of some previous studies [4-9]. Green propolis, a specific kind of Brazilian propolis, reduced both neuronal necrosis and apoptosis of retinal ganglion cells [17,19]. Red propolis had a similar anti-inflammatory effect in another experimental model of a corneal chemical burn [10]. Furthermore, its enhancement of wound healing is associated with a decline in free radical generation, which provides a partial protection against oxidative stress induced lipid peroxidation [19].

Mechanical deepithelization usually requires less time for wound closure than those caused by an alkali burn [20,21]. Such a difference is due to the fact that a severe alkali burn elicits stromal dysregulated inflammation and scar formation through inducing immune cell infiltration and myofibroblast formation from keratocytes [22-24]. This type of inflammatory response is not self-limiting and is associated with oxidative damage, causing degradation of the stromal matrix [20,25]. These pathological findings were the basis for our choice of the AgNO3 alkali burn model to evaluate the effects of propolis on corneal wound healing.

Complete re-epithelization occurred in different eyes between 48 and 120 h although the exact mean re-epithelization time was not determined. It seemed closer to 48 than to 120 h, because at 48 h wound closure in the VH and BP groups was already 81% and 92%, completed, respectively. This time requirement for healing is consistent with other reports cited previously. Epithelial cell renewal is dependent on basal cell mitotic activity. These proliferating cells are derived initially from quiescent stem cells in the limbal periphery, which are activated by injury to become proliferative [26,27]. We only evaluated regenerative capacity in the presence and absence of BP based on Ki67 staining in the proliferating basal cell layer. In the BP group especially at 12 h there was more Ki67 staining than in the VH group.

Acute inflammation induced by an alkali burn was evaluated by counting the total number of infiltrating neutrophils in the central injured region. At 12 h, in both groups, neutrophils counts increased. As the number of neutrophils in the cornea of rats is small under normal conditions [28,29], the increase in neutrophil number during the first 12 h is possibly due to neutrophil infiltration from the periphery. BP containing eye drops did not prevent or inhibit their inward migration during this period. At 24 h, their numbers in both groups increased further by more than 100%. However, in the VH group the neutrophil count values were 25% higher than those in the BP group. This difference suggests that BP has significant anti-inflammatory effects in this model. Moreover, this difference persists even after 48 h. At 120 h, the neutrophil count had declined by about 80% from that at 48 h. There no longer was any difference between their numbers in the BP and VH groups. These time-dependent transient increases in neutrophil stromal counts are in accord with those shown in other studies [30-32]. Thus, BP did not inhibit or change the time-course required for completing wound closure even though acute inflammation was partially suppressed during a portion of the healing response.

In summary, the present work suggests that BP reduces the inflammatory response to injury, but the overall effect of this suppression was not adequate to hasten epithelial wound healing. Although other preparations have had similar effects in pre-clinical studies and randomized clinical trials, these therapeutic options require further exploration [31,33-35]. This need is indicated by the fact that even though anti-inflammatory drugs such as corticosteroid eye drop formulations reduce local inflammation, they can contain vehicles and distinct stabilizing compounds, which may impair wound healing process.

The proposal of BP as a topical treatment of chemical burns, and potentially other aseptic keratitis, is based on both its cicatrizing and anti-inflammatory effects. It is still not clear which components of BP are responsible for these effects, but some studies have already identified effective anti-inflammatory constituents [36,37]. Combining these BP actions, it has certain pro-healing attributes not found in other anti-inflammatory medications (e.g. corticosteroids) currently available for the treatment of corneal injury.

## Conclusions

Topically applied BP accelerated wound healing and reduced inflammatory response after silver nitrate-induced alkali burns in rats. The clinical usefulness of a treatment with topical BP in ocular surface diseases associated with corneal epithelial defects should be explored further as an alternative for the prevention of these potentially blindness conditions.

## Competing interests

Financial support: São Paulo Research Foundation - FAPESP.

Financial interest statement: The authors have no financial competing interests in this study.

## Authors’ contributions

LM carried out the experiments, participated in the statistical analysis and drafted the manuscript. JP: participated in the design of the study, as well as the statistical analysis and drafted the manuscript. ER participated in the design of the study and revision of the manuscript. SG carried out the histology and immunohistochemistry analysis. All authors read and approved the final version of the manuscript.

## Authors’ information

SG has been studying, in contribution with other authors, the effects of Brazilian propolis in different tissues. As an associate professor and a former chief of the Department of Pathology of the University of São Paulo, he carried out the histology and immunohistochemistry analysis of multiple studies, which correlated Brazilian propolis with inflammation and wound healing in several tissues, including skin and bones.

JP and ER are associate professors of ophthalmology in the Department of Ophthalmology, Otorhinolaringology, Head and Neck Surgery of the University of São Paulo, and they both have been working with experimental corneal models, related to ocular surface disease in dry eye syndrome, and other ocular inflammation conditions. JP was a former chief of the Division of Ophthalmology of this Department, and ER is the current chief of the same Division.

LM is a young ophthalmologist who had been working with SG since medical school, before ophthalmology residency. After finishing residency of ophthalmology and a one-year fellowship in cornea, he was invited by those professors to develop the present study, as part of his PhD.

## Pre-publication history

The pre-publication history for this paper can be accessed here:

http://www.biomedcentral.com/1472-6882/13/337/prepub
